# Photocatalytic Transformation of Biomass and Biomass Derived Compounds—Application to Organic Synthesis

**DOI:** 10.3390/molecules28124746

**Published:** 2023-06-13

**Authors:** Mario Andrés Gómez Fernández, Norbert Hoffmann

**Affiliations:** CNRS, Université de Reims Champagne-Ardenne, ICMR, Equipe de Photochimie, UFR Sciences, B.P. 1039, 51687 Reims, France

**Keywords:** organic synthesis, photocatalysis, platform chemicals, reaction mechanisms, renewable resources, sustainable chemistry

## Abstract

Biomass and biomass-derived compounds have become an important alternative feedstock for chemical industry. They may replace fossil feedstocks such as mineral oil and related platform chemicals. These compounds may also be transformed conveniently into new innovative products for the medicinal or the agrochemical domain. The production of cosmetics or surfactants as well as materials for different applications are examples for other domains where new platform chemicals obtained from biomass can be used. Photochemical and especially photocatalytic reactions have recently been recognized as being important tools of organic chemistry as they make compounds or compound families available that cannot be or are difficultly synthesized with conventional methods of organic synthesis. The present review gives a short overview with selected examples on photocatalytic reactions of biopolymers, carbohydrates, fatty acids and some biomass-derived platform chemicals such as furans or levoglucosenone. In this article, the focus is on application to organic synthesis.

## 1. Introduction

The chemical transformation of biomass as a renewable feedstock as well as catalysis play a key role in sustainable chemistry [1]. In many cases, biomass is also an important alternative to fossils in the context of international economic and political dependencies and conflicts. In the context of chemical valorization, it should be pointed out that the molecular structure of biomass is different form corresponding fossil carbon compounds [2,3,4]. For example, carbohydrates which represent 75% of the biomass have a higher oxygen contain [5,6]. Also, lignin (about 20% of the biomass), which is the essential biomass-based source for aromatic compounds, contains higher amounts of oxygen [7]. The presence of numerous functional groups in such compounds makes them particular interesting for the production of new platform chemicals [8,9,10,11,12] for industry and also for many applications to fine chemistry and organic synthesis [13,14,15,16,17,18,19,20]. Catalysis is a further key element of green chemistry [21]. The catalytic transformation of biomass or biomass-derived platform or fine chemicals thus permits synergistic effects of sustainable chemistry as two requirements of green chemistry [1] are combined [22,23]. 

Photochemical reactions have been recognized as being at the origin of sustainable chemistry [24,25,26,27,28]. They also play an important role in organic synthesis [29,30,31,32]. In this context, photochemical reactions also became very interesting in the chemical or pharmaceutical industry [33]. Applications of photochemical reactions in heterocyclic chemistry are particularly interesting [34,35,36]. Heterocycles are the partial structure of numerous bioactive compounds and play an important role in material science, for example for organic semi-conducting materials. More recently, photocatalytic reactions have gained an enormous interest in organic synthesis in the academic domain as well as in industry [33,37]. Various kinds of photocatalysis are reported [38]. They are characterized by different elementary steps such as sensitization by energy transfer, electron transfer or hydrogen transfer. The latter are often coupled and described as proton-coupled electron transfer (PCET) [39,40,41]. In particular, photocatalysis with visible light is now investigated [42,43]. The systematic study of these reactions opens perspectives for organic synthesis [44,45]. The generally mild photochemical reaction conditions facilitate a large number of C-H activation processes. Additionally, classical thermal reactions such as the Diels–Alder reaction can be facilitated [46]. Often in these cases, the reaction mechanism changes. A further important aspect is the combination of photoredox with other kinds of catalysis [47,48,49,50,51,52] such as enzyme [53,54,55,56,57], organocatalysis [58,59,60,61,62] or with conventional organometallic catalysis [63,64,65,66,67,68]. All these forms of catalysis are currently applied to the transformation of biomass or biomass-derived platform chemicals.

Further synergies in connection with sustainable chemistry are currently developed by systematic investigations of photocatalytic transformations of lignocellulosic material and platform chemicals obtained from biomass. Various aspects of this domain have been recently reviewed [69,70,71]. Therefore, the present article mainly focuses on the application of such strategies in fine chemistry or organic synthesis. 

## 2. Photocatalytic Transformations of Biopolymers

By far most of the biomass is composed of polymers. Polysaccharides and lignins are the most important compound families. In many cases, photocatalytic transformation of such material yields mixtures of monomers [9,69,72,73]. For example, the depolymerization of cellulose is photo-catalyzed by TiO_2_ [71,74,75]. Reactive radical species are generated, which further react with oxygen. Under acidic conditions, such a catalysis may support the formation of furan derivatives, which are important platform chemicals. In a similar way, the photochemically supported Fenton oxidation has been used for depolymerization of polysaccharides [76]. 

**Figure 1:**
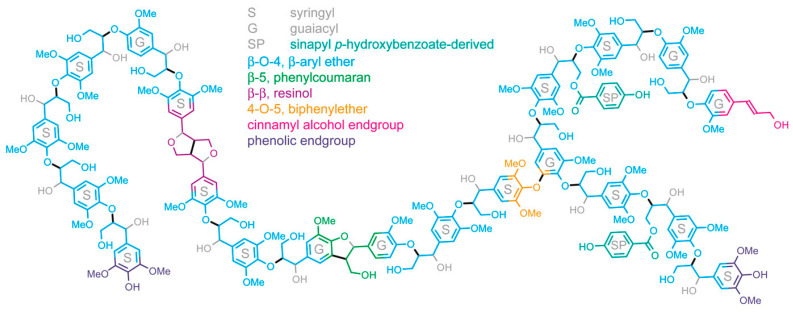
Structure of lignin. (Reproduced with the permission of the American Society of Plant Biologists [81]).

**Scheme 1:**
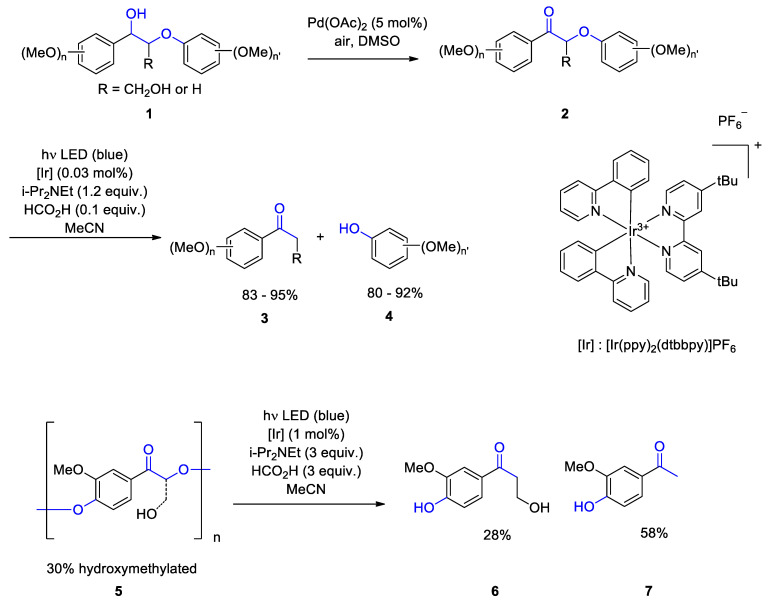
Catalytic cleavage of lignin model dimers and polymers involving photoredox catalysis with visible light.

Similar photoredox catalytic reactions have been carried out with lignin [77,78,79,80]. Lignin is an important renewable source for aromatic compounds (Figure 1) [7,81]. For example, production processes of vanillin from lignin are frequently studied [82]. Another research approach with these material deals with the study of photochemical reactions [83]. Studies on the depolymerization with processed or native lignin are particular challenging. In order to obtain information on possibly efficient elementary steps, reactions of partial structures of lignin are investigated. Using Pd catalysis lignin fragments such as **1** are selectively oxidized to the corresponding acetophenone derivatives **2** (Scheme 1) [84]. Primary alkyl alcohol functions are not transformed under these conditions [85]. In a second photoredox catalytic reaction, C-O bond cleavage takes place yielding the acetophenone derivatives **3** and phenols **4**. The iridium complex [Ir(ppy)_2_(dtbbpy)]PF_6_ was used as photoredox catalyst. The first process was separately performed and a workup without purification was carried out. This was necessary because different solvents were used in both steps. The resulting raw material was subjected to the photoredox catalytic step. The photoredox catalytic step was also carried out under similar conditions with model oligomers or polymers of lignin such as **5**. The corresponding monomers **6** and **7** were isolated in high yields. 

**Scheme 2:**
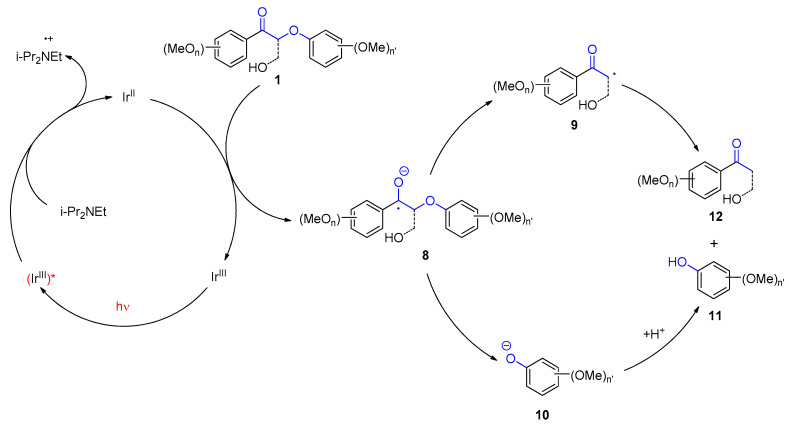
Mechanism of the photoredox catalytic cleavage of lignin model dimers.

The following mechanism has been discussed for the photoredox catalytic part (Scheme 2). After excitation of the [Ir(ppy)_2_(dtbbpy)] photocatalyst, which is an Ir^III^ species, electron transfer from the sacrificial amine donor occurs and an Ir^II^ species is generated. The latter is capable of reducing the acetophenone derivative **1**, which leads to a radical anion **8**. Fragmentation yields a neutral radical **9** and a phenolat **10**. Protonation yields the final product **11**. The neutral electrophilic radical undergoes hydrogen abstraction of hydrogen atom transfer (HAT), yielding the final acetophenone derivative **12**. This step may also involve electron transfer followed by proton transfer [86]. Two catalytic processes are performed consecutively in the present transformation. 

This transformation was also carried out with sunlight. Similar transformations of small model compounds have been reported [87,88,89]. Such studies have also been performed in the context of the sustainable production of vanillin [90,91]. In the context of organic photochemical reactions, for example cycloaddition with alkenes, derivatives of this compound are particularly interesting because in such transformations a high degree of molecular complexity and diversity is generated without chemical activation and thus reducing waste production [27,92,93,94].

## 3. Carbohydrates

**Scheme 3:**
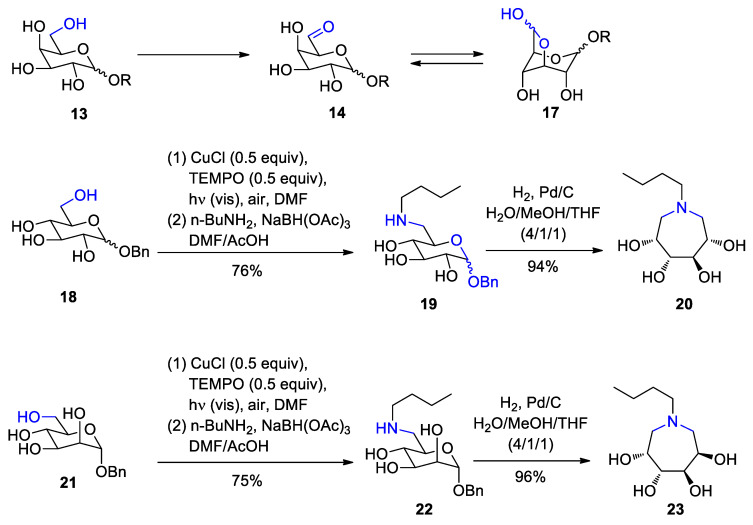
Convenient synthesis of tetrahydroxyazepanes form glucose and mannose derivatives.

**Scheme 4:**
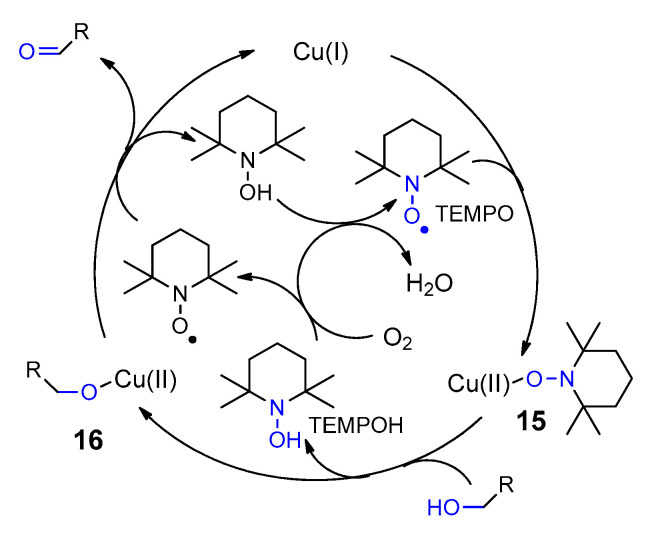
Mechanism of the Semmelhack reaction.

Carbohydrates are versatile platform compounds and synthons in organic synthesis. As enantiomerically pure compounds they or their derivatives are key intermediates in asymmetric synthesis [95,96]. The systematic arrangement of chiral centers is particularly interesting in this regard [97,98,99,100]. Photocatalytic reactions have been applied in this domain [101]. In the present example, the selective oxidation of the primary alcohol function (**13**) into an aldehyde (**14**) plays a key role (Scheme 3) [102]. No secondary functions should be oxidized. Thus many syntheses with carbohydrate synthons can be facilitated since the number of protecting and deprotecting steps is reduced. The reaction is well known and can be carried out with galactose oxidase and molecular oxygen [103]. Since this enzyme activity is limited to galactose derivatives, various biomimetic strategies have been developed in order to extend the reactivity to further hexoses such as glucose or mannose derivatives [104,105,106,107]. Aside other methods, the Semmelhack reaction [108] can be used for the oxidation of primary alcohols to aldehydes [109]. Molecular oxygen is used as oxidant and copper salts and TEMPO (2,2,6,6-Tetramethylpiperidinyloxyl) are used as catalysts or as co-catalysts. Cu(II) acts as an oxidant (Scheme 4) [110]. This species (**15**) is generated by the addition of TEMPO and release of TEMPOH. In this ligand exchange process, the primary alcohol is added (**16**). The release of the carbonyl product regenerates the Cu(I) catalyst. The co-catalyst TEMPO is regenerated by the oxidation of TEMPOH by molecular oxygen. It has been observed that when the transformation is carried out with visible light irradiation, the reaction becomes efficient and can be applied to organic synthesis (Scheme 3) [102]. This effect can be explained by the fact that the Cu-O bond is weakened when such complexes are photochemically excited via a ligand to metal charge transfer (LMCT). Thus, ligand exchange steps in the mechanism are accelerated [111]. The oxidation product is stabilized by the formation of an anhydro derivative such as **17**. Isolation and purification of these products were difficult. However, in situ transformation by a reductive amination efficiently yielded the corresponding glucose and mannose derivatives **19** and **22**. Elimination of the benzylic protecting group followed by an intramolecular reductive amination yields the tetrahydroxyazepanes **20** and **23**. Using the enzyme catalytic oxidation with galactose oxidase, tetrahydroxyazempanes with glucose- or mannose-related configurations are not accessible. Tetrahydroxyazepanes are azasugars. Such compounds possess various biological activities such as the inhibition of glycosidases and glycosyltranferases or anti-cancer activities [112,113,114]. In the present synthesis many steps of protecting and deprotecting different hydroxyfunctions and the utilization of corresponding reagents are avoided. As the irradiation was performed with visible light with visible light, the use of sunlight can be projected [28,115].

**Scheme 5:**
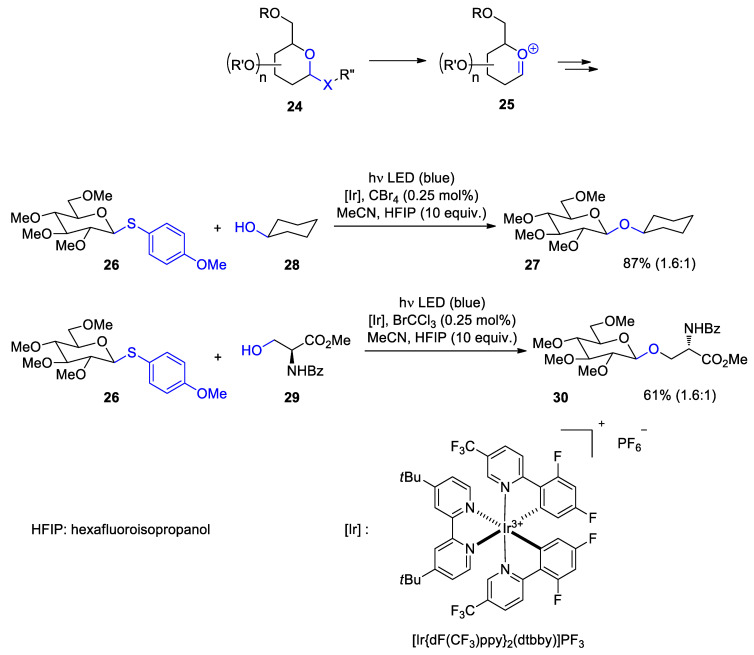
Photoredox catalytic glycosidation with anisylthioether derivatives. β-Anomeric glucosilation products are preferentially formed.

**Scheme 6:**
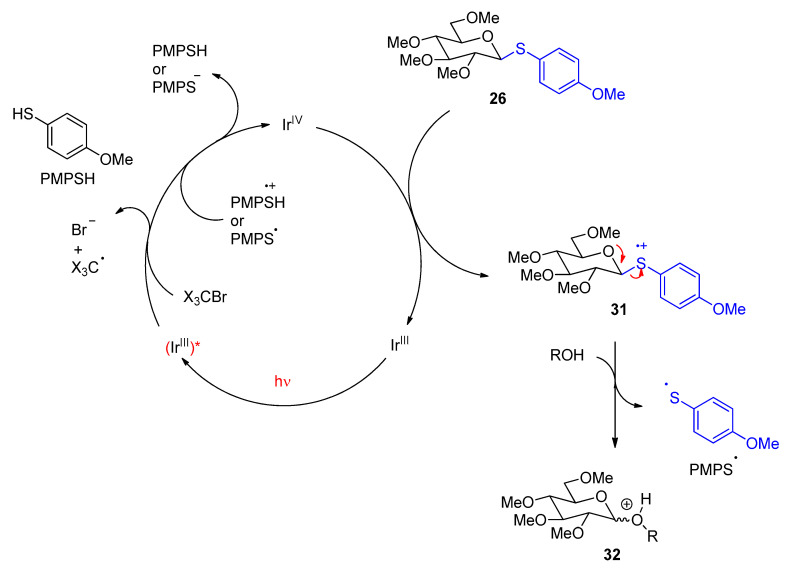
Mechanism of the Photoredox catalytic glycosidation with anisylthioether derivatives involving photochemical electron transfer with an iridium complex.

Glycosylation plays a central role in carbohydrate chemistry. During recent years various photocatalytic methods have been developed for this purpose [116,117]. Many of these reactions are carried out with visible light and with corresponding LED’s as light source. Such reaction conditions are particularly mild and energy consumption is low. Carboxonium intermediates **25** play an essential role in such transformations (Scheme 5) [118,119]. These species are generated from precursors **24** with corresponding leaving groups. Glycosylation then occurs by a nucleophilic attack. As these intermediates carry a positive charge, protonating agents or Lewis acidic compounds are often used as promotors. Oxidation such as photochemical electron transfer induced release of the leaving group is an alternative [120]. In this context, photoredox catalytic reactions have been systematically studied. Thus, compound **26** carrying an electron rich thioanisyl as the leaving group at the anomeric center was efficiently transformed with **28** into the corresponding glycosylation product **27** (Scheme 5) [121]. Additionally, more complex compounds such as the serine derivative **29** were successfully transformed. In most cases, the formation of β-anomers was favored. Tetrahalogenmethanes were added to the reaction mixture. They play a key role in the initiation step of the catalytic cycle. The following mechanism has been discussed. The reaction starts with the photochemical excitation of the iridium photocatalyst (Scheme 6). The Ir^III^ species is oxidized by a tetrahalogen methane. The resulting Ir^IV^ complex is reduced by the anisylthioether function (PMPS) in **26**. The photocatalyst is thus regenerated. After the release of the PMPS radical from **31** and the nucleophilic, the attack of the alcohol compound takes place (**32**). The photochemically excited Ir^III^ complex can also be oxidized in the reaction with intermediates of the PMPS leaving group. As it has already been pointed out, acidic activation of a leaving group leads to the formation of the carboxonium ion intermediate **25** (Scheme 5). Electronic excitation can significantly increase the acidity [122,123] and thus induce the release of a leaving group [124]. Thus, the trichloroimidate group was activated by photochemically excited phenols or naphthols [125]. Thiourea derivatives have also been used for the same purpose [126]. In some cases, both electron and proton transfer processes are involved in this activation [127].

**Scheme 7:**
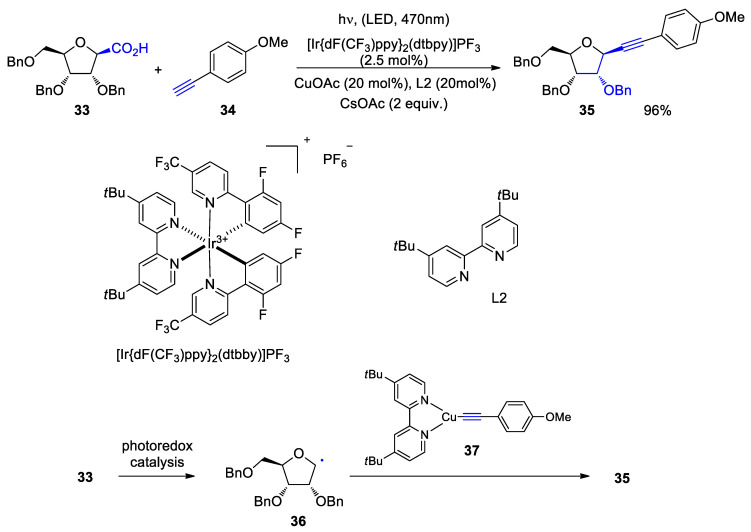
Combined photo and copper catalysis applied to the coupling reactions of carbohydrate derived acids with alkynes.

C-Glycosids play an important role as bioactive compounds [128]. In such compounds, an acetal C-O bond is replaced by a more stable C-C bond. Thus, many of these products possess enzyme inhibitor properties. For example, glucosidases or many nucleoside transformations can be inhibited. In the latter case, a C-N bond is replaced by a C-C bond [129]. In the reaction of the 1-β-d-glucofruanose derivative **33** with *p*-methoxyphenylacetylene **34**, only one diastereoisomer of the C-glucosid **35** was formed (Scheme 7) [130]. The photoredox catalytic part is carried out with an iridium catalyst. Compound **33** is decarboxylated and the intermediate **36** is formed. Such photodecarboxylation reactions are now frequently studied [131]. In the copper catalytic cycle, the complex **37** is formed, which reacts with the radical species **36** leading to the final product. The copper catalysis part is activated by addition of the ligand L2. In this case organic photocatalysts were less efficient. It cannot be completely excluded that the copper catalytic step is not affected by light absorption. In the present report, 28 C-glucosides have been synthesized with yields up to 96%. Heteroaromatic and alkyl acetylene derivatives have also been added to furanosyl radicals.

**Scheme 8:**
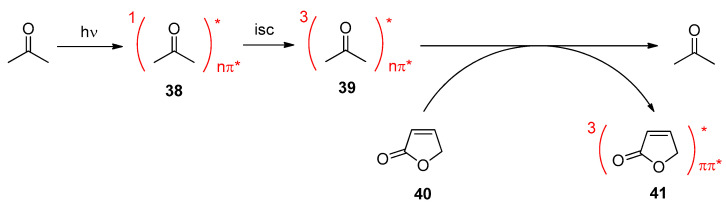
Electronic excitation of a furanone to the triplet state via triplet energy transfer.

Chemical reactions can efficiently be induced by photosensitization. In this case, a chemical compound absorbs light and transfers its excitation energy to the substrate, which is electronically excited in this way. The latter then undergoes a photochemical reaction. The ground state of photosensitizer is regenerated and a new sensitizing process may take place. The photosensitizer acts as catalyst [38]. Often ketones are used as photosensitizer that efficiently transfer their triplet energies to a substrate. For example, this technique is applied to the photochemical transformation of α,β-unsaturated lactones. Often, these compounds possess a high excitation energy and low wavelength light irradiation is necessary for direct excitation to the S_1_ state. Such conditions leads to unselective reactions. Since triplet energies are lower, triplet energy transfer from a sensitizer often leads to more selective reactions. More convenient reaction conditions such as room temperature instead of low temperature can be applied. Such a sensitization of a α,β-unsaturated butyrolactone or furanone is depicted in Scheme 8. Acetone absorbs light and is transferred to its singlet state S_1_ **38** possessing nπ* character. After intersystem crossing to the corresponding energy lower triplet state, T_1_ **39** is reached. Triplet energy transfer now occurs to the furanone **40** that is thus transferred to its triplet state T_1_ **41** with ππ* character. The latter species undergoes photochemical reactions. Acetone also absorbs at lower wavelengths (λ_max_ (nπ*) = 278 nm) [132]. However, irradiation at longer wavelengths such as 300 nm generates sufficient excited molecules for sensitization when acetone is used as a solvent or co-solvent. 

**Scheme 9:**
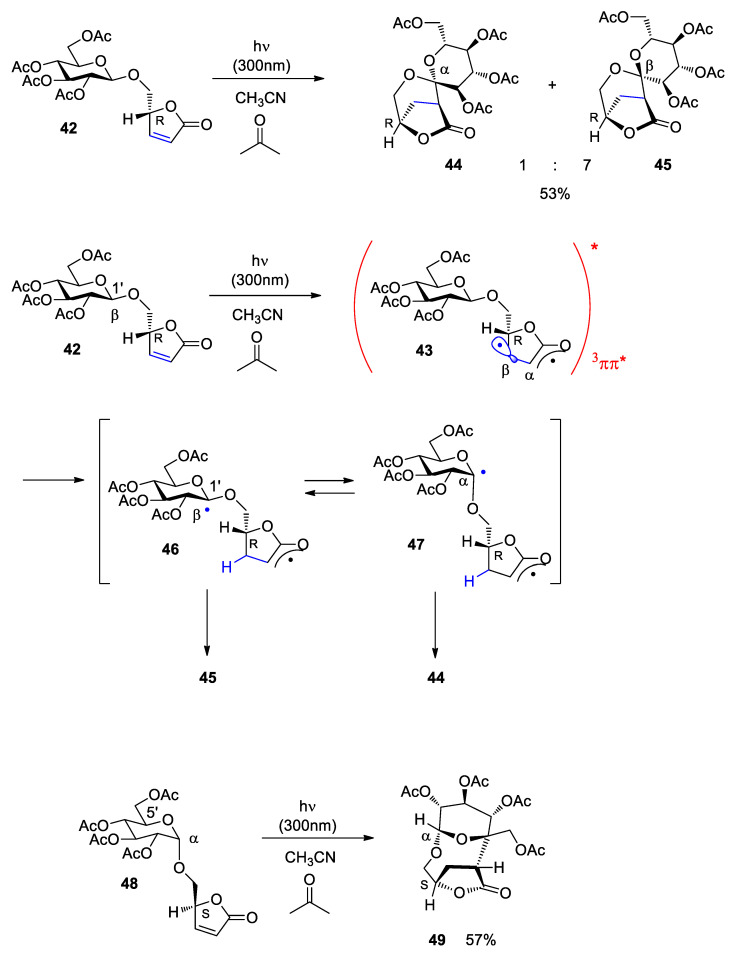
Intramolecular photosensitized radical addition of a carbohydrate moiety to a furanone. Acetone was used as sensitizer and cosolvent in a ratio of 1/4 with acetonitrile.

Under these conditions, furanone compounds such as **42** carrying a glucosyl moiety have been electronically excited to their triplet state **43** (Scheme 9) [133]. Sugar derivatives with their secondary alcohol functions are excellent hydrogen donors for hydrogen abstraction or hydrogen atom transfer (HAT) processes [134,135]. Such reaction steps are also observed in non-catalytic photochemical transformations. For some examples, see refs. [136,137,138]. Hydrogen abstraction is observed with many triplet exited compounds [94,135,137,139,140]. In the photosensitized reaction of glucosylated furanone derivative **42**, the spirocyclic compounds **44** and **45** are formed. In contrast to many conventional radical addition reactions of such α,β-unsaturated lactones, a C-C bond is formed in the α-position. The electronic excitation occurs via triplet energy transfer from acetone to **42**. The ^3^(ππ)* state (**43**) is characterized by the suppression of the C=C π-bond and an increased spin density in the β-position [135,141]. For this reason, hydrogen abstraction occurs from the anomeric center of the sugar moiety into this position and the intermediates **46** and **47** are formed, both in conformational equilibrium. Radical combination yields the final products **44** and **45**. In this reaction, the regioselectivity of hydrogen abstraction depends on the relative configuration of the chiral centers in the anomeric position and the chiral center at the furanone moiety. When the corresponding α-anomer diastereoisomer **48** is transformed under the same conditions, hydrogen abstraction occurs at the position 5′ of the glucosyl substituent and the macrocyclic product **49** is formed. Similar reactions have been carried out with direct excitation of a furanone moiety to the singlet state and subsequent intersystem crossing to the triplet state. The transformation under these conditions needed lower wavelength irradiation and low reaction temperature [142]. It should further be pointed out that for the triplet sensitized of C-H activation, no particular reagents are needed, which often leads to the production of additional waste. Furthermore, acetone is used as a co-solvent and no chromatographic separation of the sensitizer or a photocatalyst is necessary. 

## 4. Fatty Acids

Fatty acids obtained from triglycerides play an important role in the chemical valorization of biomass. Their use for the production of surfactants belong to the basic culture techniques of humanity. More recently their application as part of biofuel is reported [143]. Fatty acid methyl esters (FAME) can be obtained by photocatalytic esterification to produce biodiesel [144,145,146].

Photodecarboxylation is an interesting transformation both for biofuel production and in organic synthesis [147,148,149] as it leads to radical intermediates that can form alkanes or further react with a variety of substrates. Decarboxylation is also carried out using photochemical electron transfer [150,151,152,153,154]. Such reaction steps also play an important role in many metal catalyzed reactions [131,150,155,156]. In this context, but also for application to organic synthesis, decarboxylation is an interesting transformation as it leads to a variety of radical intermediates. 

Hatanaka et al. have transformed palmitic acid into the corresponding *n*-pentadecane [157]. This method consists of the photoelectron transfer of the carboxylate with a photogenerated cation radical of phenantrene followed by decarboxylation. The hydrogen abstraction from a thiol results in the formation of the corresponding alkane. Nicewicz et al. used of an acridinium photooxidant in the presence of an organic base for a decarboxylation [158]. The mechanism involves a photochemical electron transfer to the acridinium from the carboxylate followed by CO_2_ release. The generated carbon radical abstracts a hydrogen from the in situ generated thiophenol. Wang et al. reported a route for photocatalytic decarboxylation for alkane production using fatty acids. Their system uses semiconductor Pt/TiO_2_ and irradiation using 365 nm LEDs, and a hydrogen atmosphere resulted to be crucial for the high yields (>90%), while several fatty acids were successfully transformed in the corresponding alkanes [159].

Enzyme-catalyzed reactions play a key role in the transformation of biomass or biomass-derived compounds. Recently, an algal photoenzyme named fatty acid photodecarboxylase (CvFAP) was discovered. This photoenzyme transforms fatty acids into the corresponding alkanes upon light irradiation [160,161,162]. Among the different uses of this photo-enzyme, several methods were reported to obtain, for example, bio-propane and bio-butane from fatty acids [163,164], and also bio-diesel [165] depending on the substrate that was used.

Concerning the application of such reactions to organic synthesis, radical intermediates are often generated by decarboxylation of carboxylic acids or their derivatives [166,167,168,169,170]. These radicals undergo addition to various acceptor molecules. In many of these reactions, photocatalysis involving electron transfer is applied. The transformations are often also carried out with corresponding fatty acids or their derivatives.

**Scheme 10:**
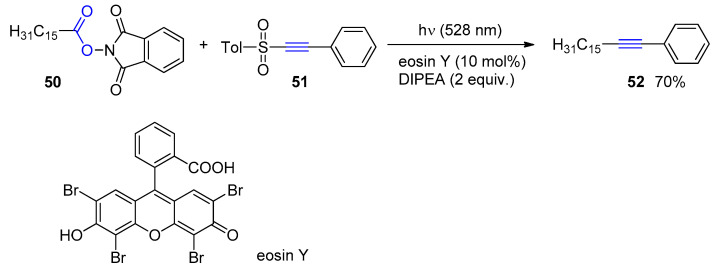
Photochemical decarboxylation of a fatty acid derivative applied to a coupling with alkynes.

To perform the photodecarboxylation of fatty acids followed by radical reactions, a strategy using an activated form of the fatty acid was used. König et al. used the N-hydroxyphtalimide activated esters such as **50** of palmitic acid to perform the photodecarboxylation reaction (Scheme 10) [171]. They used 10 mol% of eosin Y, an organic and unexpensive sensitizer, and irradiation with green light (λ = 528 nm) to start the photodecarboxylation, and the resulting radical underwent coupling with alkynes such as **51** carrying a sulfone group. Diisopropylethylamine (DIPEA) was used as an electron donor in the photoredox catalytic process. The same reaction was successfully carried out with corresponding derivatives of lauric acid, myristic acid or stearic acid. Corresponding derivatives of unsaturated acids such as oleic or linoleic acid were also efficiently transformed into the corresponding coupling products. Glorius et al. [172] reported the C-H alkylation of heteroarenes using aliphatic carboxylic acids. Their method used an iridium photocatalyst in 0.5 mol% to perform the process. The group tolerance is large and two fatty acids were also used in this transformation.

**Scheme 11:**
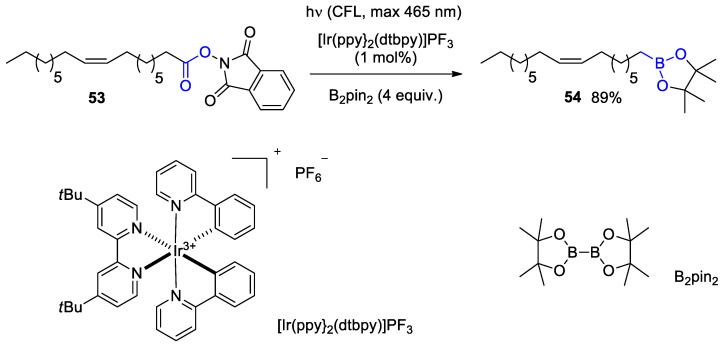
Photoredox catalytic borylation of a fatty acid via photocatalytic decarboxylation.

**Scheme 12:**
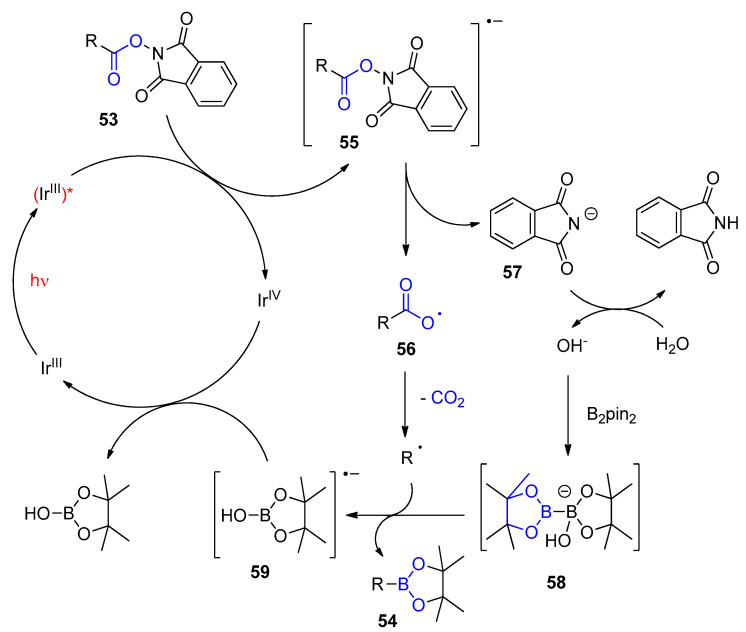
Suggested mechanism of the photoredox catalytic borylation with fatty acids (Scheme 11).

Photodecarboxylation can also be used to further functionalize organic molecules; for example, a decarboxylative borylation was described by Li et al. to obtain the corresponding alkyl boronates (Scheme 11) [173]. They performed the reaction using the activated N-acyloxyphtalimides such as **53** obtained from oleic acid. Irradiation using visible light and an iridium photocatalyst resulted in the photodecarboxylation and the radicals were successfully trapped by a B_2_pin_2_ derived intermediate to obtain the corresponding borylated product **54**. The photoredox catalysis mechanism depicted in Scheme 12 has been suggested. The photochemically excited Ir(III) catalyst is able to reduce the N-acyloxyphtalimide **53** yielding the radical anion **55** and an Ir(IV) species. Fragmentation of this intermediate yields the carboxyl radical **56**, which immediately releases carbon dioxide. Concomitantly, the phthalimide anion **57** is formed. Protonation of the latter by water leads to hydroxide ions, which react with B_2_pin_2_ (**58**). Reaction of **58** with the alkyl radicals (R**^·^**) yields the final product **54**. The boron ester-derived radical anion **59** is oxidized by the Ir(IV) species, thus regenerating the photocatalyst. 

The same reaction was also successfully carried out with a musristic acid derivative leading to a corresponding borylated product. In a modified procedure, corresponding derivatives carrying a BF_3_K group have been synthesized. In a different report, Aggarwal et al. [174] reported the conversion of the same activated N-acyloxyphtalimides into boronic esters by simple irradiation with blue LEDs, no additional additives or metal catalysts were needed in this case. The photogenerated radicals were trapped by bis(catecholato)diboron. In this report, among the several substrates used, three fatty acids were used with yields up to 90%.

**Figure 2:**
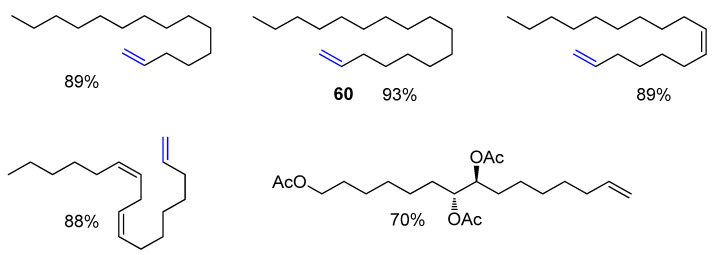
Terminal alkenes obtained by decarboxylation of fatty acids. Combined photoredox and metal catalysis was applied.

Glorius et al. have efficiently transformed N-(acyloxy)-phthalimides of fatty acids such as **50** (Scheme 10) into corresponding terminal alkenes (Figure 2) using photocatalysis with an organic photocatalyst (dihydrophenazine derivative) combined with copper catalysis [175]. Compound **60** was synthesized on gram-scale.

**Scheme 13:**
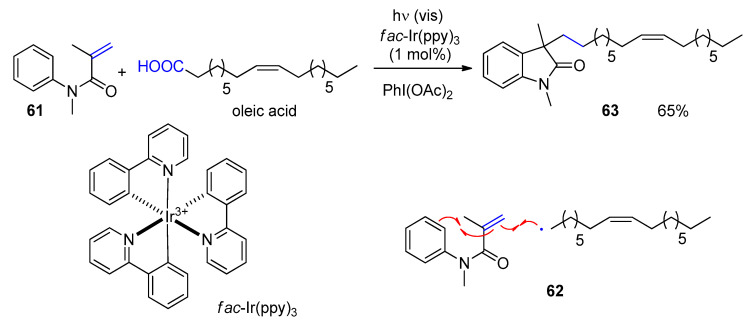
Photoredox catalytic addition cyclisation reaction with radicals generated from oleic acid.

Radicals generated by decarboxylation of fatty acids are also involved in complex radical tandem reactions. Such a reaction with oleic acid has been described by Zhu et al. (Scheme 13) [176]. Radicals are generated from this acid involving photoredox catalysis with visible light and, an iridium catalyst and PhI(OAc)_2_. Michael addition to the metacrylanilide **61** and cyclization (**62**) yields a quaternary oxindole **63**. In the present example, two C-C bonds are formed under the same photocatalytic conditions, thus avoiding a further activation.

## 5. Biomass-Derived Platform Chemicals

Levoglucosenone is an interesting platform chemical, which is obtained from pyrolysis of cellulose under acidic reaction conditions [177,178,179]. Recently, it gained additional interest as a precursor of a biobased dipolar solvent [180]. Levoglucosenone is now produced on the industrial scale. Different functional groups are localized on a relatively small enantiomerically pure molecule. Thus, it becomes a very attractive synthon for organic synthesis [181,182,183,184,185]. Levoglucosenone can also be transformed into further platform chemicals such as furanone derivatives [186,187]. 

**Scheme 14:**
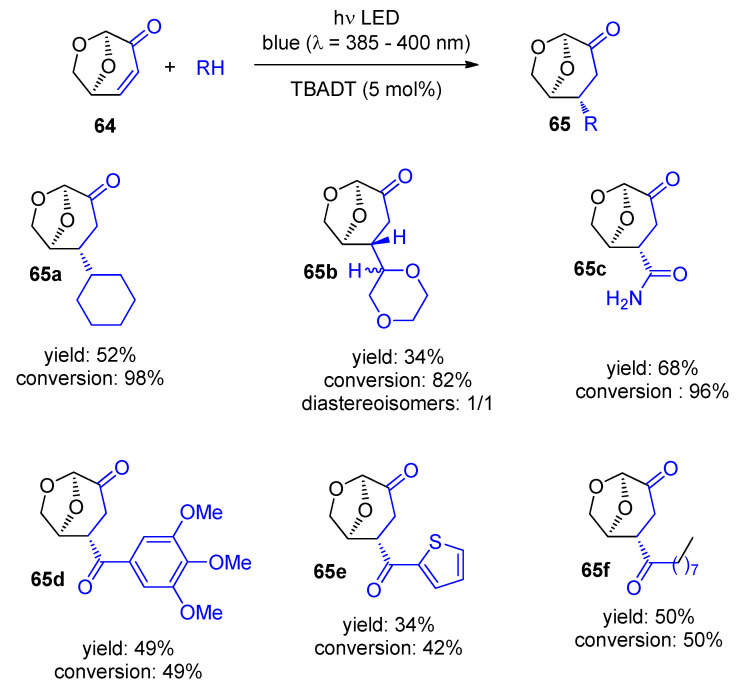
Photocatalytic addition of radicals to levoglucosenone **64**.

**Scheme 15:**
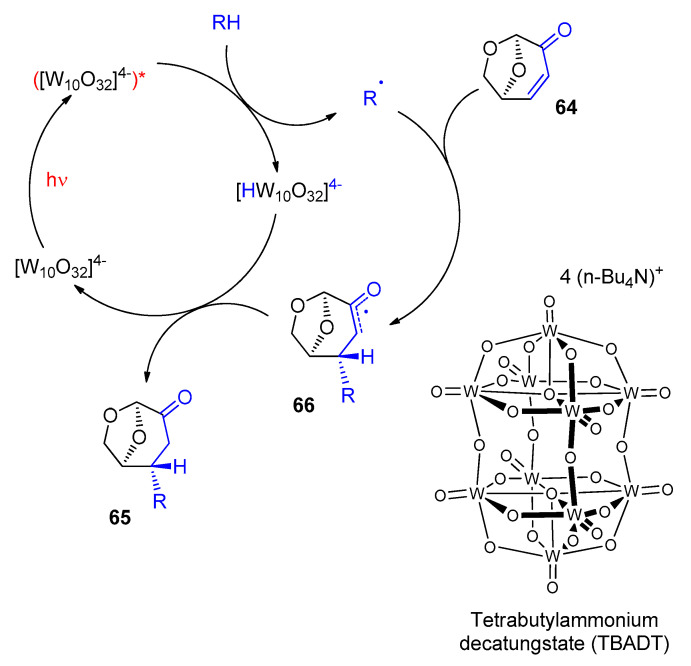
Mechanism of the photocatalytic addition of radicals to levoglucosenone **64**.

Photocatalytic reactions have also been performed with this compound. Using tetra-n-butylammonium decatungstate (TBADT) as a photocatalyst, various radical species have been added to levoglucosenone **64** (Scheme 14) [188]. Thus, alkanes (**65a**) or cyclic ethers (**65b**) have been added. The addition of formamide (**65c**) was particular efficient. The method was also used to the addition of aromatic (**65d**), heteroaromatic (**65e**) and aliphatic aldehydes (**65f**). The scope for the addition of aldehydes is large [189]. From these precursors radical intermediates are generated by hydrogen atom transfer (HAT) to the photochemically excited TBADT (Scheme 15) [190,191,192,193]. The radicals add stereospecifically *anti* with respect to the (-CH_2_-O-) moiety in **64**. The energy difference for both diastereotopic attacks of the radical intermediates is 5 kcal∙mol^−1^. The configuration of further chiral centers generated in the reaction as in **65b** was not controlled. The electrophilic radical intermediate **66** is reduced by [HW_10_O_32_]^4−^, which leads to the final product **65**. In the context of sustainable chemistry, it should further be mentioned that such reactions have been carried out with sunlight and under very simple apparatus conditions [191].

Furans are valuable platform chemicals obtained from carbohydrates [194,195,196]. They are formed by the dehydration of sugars. Thus, dehydration of pentoses or hemicellososes yields furfural while corresponding transformations of hexoses lead to hydroxymethylfurfural (HMF). These compounds are currently studied in connection with the production bulk products such as new polymers [194,197] or surfactants [198,199,200].

**Scheme 16:**
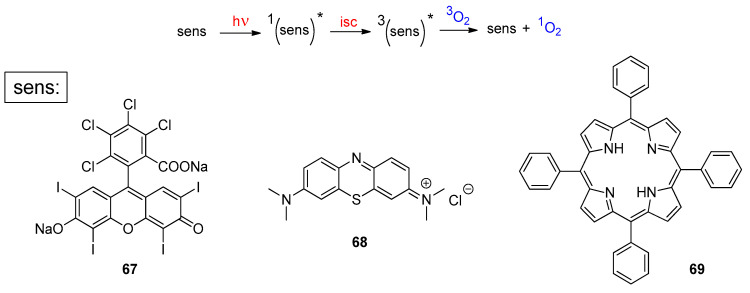
Generation of singlet oxygen involving photochemical sensitization.

One of the most-used photocatalytic reactions of furans is the photooxygenation of these compounds involving singlet oxygen. Oxygen is one of the rather rare molecules possessing a triplet multiplicity at the ground state [201,202]. The reactivity of both oxygen species is significantly different. While singlet oxygen undergoes preferentially polar reactions, for example with alkenes, triplet oxygen is characterized by its radical reactivity [203,204,205]. Most conveniently singlet oxygen is produced by photosensitization or photocatalysis (Scheme 16). In this case, a sensitizer is photochemically excited, generally to the singlet state. After intersystem crossing (isc) to the triplet state, energy transfer to triplet oxygen occurs by interaction of two species with the same spin multiplicity. The sensitizer returns to the singlet ground state and the oxygen molecule is excited to the singlet state. As the excitation energy of oxygen is rather low (23 kcal∙mol^−1^), dyes absorbing visible light such as rose bengale **67** or methylene blue **68** are often used. These dyes are soluble in protic solvents such as alcohols. For transformations in un-polar solvents, tetramethylporphyrine **69** is often used. Additionally, coordination compounds such as ruthenium complexes or nanoparticles as they are used for photoredox catalysis can be used for singlet oxygen production [206,207]. Oxidations with singlet oxygen generally occur under particularly mild reaction conditions and often with high selectivity and yields. Thus, numerous applications to organic synthesis have been reported [208]. Recently, an industrial process has been developed for the production of artemisinin where the photooxygenation is used as a key step [209]. This compound plays an important role in the treatment of malaria [210,211]. In this natural compound, a trioxane moiety is the pharmacophore. The photooxygenation of allylalcohols has been used to synthesize structural analogues [212,213,214]. Terpenes represent an important biomass-derived compound family [215,216,217,218]. The photooxygenation of these compounds is also widely applied, for example, to the industrial production of fragrances and flavors [219,220] as well as to the preparation of many other compounds [208,221,222]. Various catalytic techniques such as heterogeneous catalysts or micro and flow reactors have been developed in order to optimize the reaction [223,224,225,226,227,228].

**Scheme 17:**
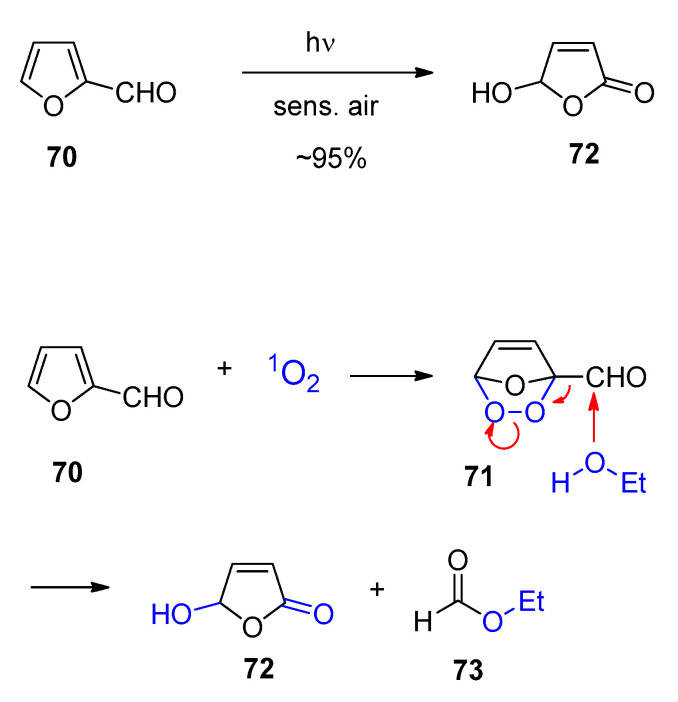
Photooxygenation of furfural **70** to hydroxyfuranone **72**.

**Figure 3:**
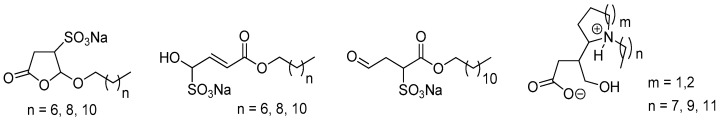
Surfactants obtained from hydroxyfuranone **72** (Scheme 17).

Furans are excellent substrates for photooxygenation with singlet oxygen [229,230]. As pointed out above, furfural **70** is a platform chemical obtained from hemicellulose or pentoses by dehydration. Its photooxygenation with singlet oxygen yields hydroxyfuranone (Scheme 17) [231,232,233]. In most cases of photooxygenation of furan derivative, an endoperoxide such as **71** is formed. As the reaction is carried out in an alcohol as solvent, this intermediate undergoes a nucleophilic attack of an alcohol molecule leading to the final products 5-hydroxyfuran-2[5H]-one **72** and a formic ester **73**. This very convenient oxidation can be carried out in the laboratory at 100 g scale [234]. Hydroxyfuranone **72** is itself a platform chemical with numerous applications to organic synthesis [235,236]. Chiral synthons can be obtained from this compound and many efficient applications to asymmetric synthesis have been reported [237,238,239,240]. At the electronically excited state, reactions are generally less stereoselective due to the suppression of the C-C π-bond [141]. Hydorxyfuranone **72** was also used for the preparation of biodegradable surfactants (Figure 3) [241]. A photoredox catalytic process was also applied for the preparation of such compounds [242,243].

**Scheme 18:**
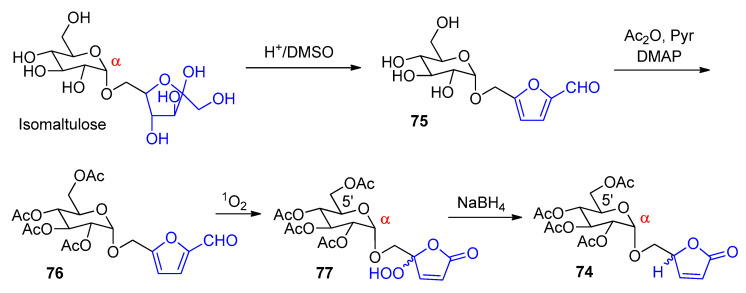
Synthesis of a α-connected glycosyl furanone **69** from isomaltulose.

Photooxygenation was also applied as a key step to the synthesis compound **74** (Scheme 18, also compare Scheme 9). Using conventional carbohydrate chemistry methodology, it is often difficult to prepare α-anomeric derivatives of hexose pyranoses such as derived from glucose for example. Isomaltulose is a disaccharide obtained by enzymatic isomerization of saccharose [244,245]. In the isomaltulose molecule, a fructose moiety in its furanose form is connected in the α-anomeric position of glucose. Selective dehydration on the fructose furanoside under acidic conditions leads to the formation a hydroxymethylfurfural derivative (**75**) [246]. Acylation (**76**) and photooxygenation yields the product **77**. Finally, reduction of the hydroperoxide **77** leads to **74** [133]. The present synthesis also provides a very convenient access to 5-hydroxymethylfuranones such as **74**. Using conventional methods of carbohydrate chemistry, these compounds are prepared with multi-step syntheses from sugars or amino acids [247,248].

## 6. Conclusions

Biomass or biomass-derived platform chemicals have become an alternative feedstock for chemical industry. They may replace conventional fossil-based resources in two senses. On one hand biomass based compounds can be used for the synthesis of already existing products in the chemical industry as they are available mainly from petroleum. On the other hand, these biomass or biomass-derived compounds can be used to produce new and original compounds, which is facilitated by characteristic structure elements such as the presence of numerous hydroxyl functions in carbohydrates. Photochemical or photocatalytic reactions make compounds or compound families available that cannot or difficultly synthesized using conventional methods of organic synthesis. A high degree of molecular complexity and diversity is thus generated in a facile way. This is explained by the complementarity of ground state and excited state reactivity. In this same context, it should be pointed out that in the past, photochemical or photocatalytic reactions of biomass-derived products have rarely been investigated in a systematic way.

Photochemical and catalytic reactions on one hand and the utilization of biomass as a renewable feedstock on the other are part of sustainable chemistry. The combination of both opens perspectives for sustainable chemistry.

## Data Availability

No new data were created or analyzed in this study. Data sharing is not applicable to this article.
